# Orthostatic hypertension—too much of a good thing

**DOI:** 10.1007/s10286-023-00961-x

**Published:** 2023-06-30

**Authors:** Italo Biaggioni, Jens Jordan

**Affiliations:** 1 Autonomic Dysfunction Center and Division of Clinical Pharmacology, Department of Medicine, Vanderbilt University Medical Center, Nashville, TN USA; 2 Institute of Aerospace Medicine, German Aerospace Center (DLR), Linder Hoehe, 51147 Cologne, Germany; 3 Medical Faculty, University of Cologne, Cologne, Germany

**Keywords:** Orthostatic hypertension, Blood pressure, Autonomic disorders, Hypertension

With assumption of the upright posture, gravitational pooling of blood and plasma extravasation in the lower body challenges the cardiovascular system. In healthy persons, activation of compensatory autonomic reflexes maintains blood pressure while standing. Failure of these compensatory mechanisms results in orthostatic hypotension, which is not only one of the most disabling symptoms in patients with autonomic failure, but is now recognized as an independent risk factor for cardiovascular morbidity and mortality [1]. At the other end of the spectrum, some people exhibit an increase in blood pressure with standing, a condition that is increasingly recognized as a risk factor for cardiovascular morbidity and mortality independent of traditional risk factors [2, 3]. A recent expert consensus panel suggested pragmatic definitions for an exaggerated orthostatic pressor response and for orthostatic hypertension [4, 5]. An exaggerated orthostatic pressor response was defined as sustained increase in systolic blood pressure by at least 20 mmHg when changing from the supine to the standing position regardless of absolute blood pressure while standing. Orthostatic hypertension was defined as an exaggerated orthostatic pressor response associated with systolic blood pressure of at least 140 mmHg while standing. However, even less pronounced changes in systolic blood pressure with standing not reaching diagnostic cutoff values for orthostatic hypotension or orthostatic hypertension may herald increased cardiovascular risk.

In this issue of Clinical Autonomic Research, Palatini et al. present data on the prevalence of orthostatic hypertension in 1245 younger-to-middle aged persons who had participated in the Hypertension and Ambulatory Recording Venetia Study (HARVEST) [6]. Study participants had to have untreated arterial hypertension, defined as an office seated blood pressure ≥ 140/90 mmHg. Participants underwent orthostatic testing at baseline and again after 2 weeks and after 3 months, which is a particular strength of the study. The authors also obtained ambulatory blood pressure recordings and urinary norepinephrine and epinephrine measurements. The authors should be commended for this important contribution to the topic.

This carefully conducted study highlights the challenges in diagnosing orthostatic hypertension. The prevalence of orthostatic hypertension as per the consensus definition [4, 5] was 0.7% when the mean of two visits was used, but no study participant met criteria for orthostatic hypertension on all three visits. The authors also obsevred that the increase in systolic blood pressure on standing was inversely correlated with supine blood pressure. Participants who exhibited a white coat effect, defined as an elevated office blood pressure with a normal 24-h ambulatory blood pressure, had a lower prevalence of orthostatic hypertension. The finding is surprising given that both conditions are likely mediated through excess sympathetic activation [7]. Another interesting finding is that the prevalence of orthostatic hypertension was greater in patients who became normotensives (2.1%, 8 out of 286) than those who remained hypertensives (0.25%, 2 out of 794). Although the interpretation of these findings is limited by the low number of observations, the finding suggests that an orthostatic pressor response in younger people, seen more in normotension than in hypertension, is a different entity than an orthostatic pressor response in older persons, which make up the majority of patients with arterial hypertension.

The study by Palatini et al. [6] reveals the gaps we still have in understanding orthostatic hypertension. Namely, what should be the diagnostic criteria and what are the implications of said diagnosis. How does the presence of orthostatic hypertension influence treatment decisions? It is important to note that the patient cohort in the study by Palatini had stage 2 hypertension according to recently updated guidelines. These patients, therefore, require treatment regardless of the presence of orthostatic hypertension, raising questions about the practical relevance of making this diagnosis. Nonetheless, other studies have shown that the presence of orthostatic hypertension is associated with negative outcomes in patients with established hypertension [2], suggesting that, perhaps, these patients should be treated more intensely. A few patients had isolated orthostatic hypertension and normal 24-h ambulatory blood pressures. Unfortunately, we lack evidence to guide clinical management decisions in these patients, such as the need to prescribe antihypertensive medications. It seems prudent to implement nonpharmacological treatment in patients with isolated orthostatic hypertension and follow their hypertension status closely.

A separate question is whether the presence of orthostatic hypertension should influence the selection of antihypertensive treatment. The few mechanistic studies suggest age-related differences; an increase in sympathetic responses has been proposed as the mechanism for orthostatic hypertension in the young and increase vascular stiffness in older patients [8, 9]. Moreover, hypovolemia may predispose to sympathetic overactivation and orthostatic hypertension [7]. It is less clear how this translates to the selection of antihypertensive treatment.

Palatini et al. propose that the threshold for diagnosing orthostatic hypertension in younger persons may have to be revisited. Indeed, lesser increases in blood pressure with standing predict cardiovascular risk in younger-to-middle aged people [10, 11]. We would argue that the current consensus limit of 20 mmHg for the definition of orthostatic hypertension may have some benefits, particularly in younger individuals with a normal blood pressure while supine or seated. In this population with isolated orthostatic hypertension, overall cardiovascular risk appears to be low. Lowering the diagnostic threshold increases the risk of overdiagnosing orthostatic hypertension, which could, in turn, lead to stigmatization and overtreatment. Suffice it to say that upright blood pressure measurements in real life are not only affected by physiological variability but also by less than optimal methodology, making a lower cutoff limit prone to innacuracies. It is possible that younger-to-middle aged populations have a lower prevalence of orthostatic hypertension because some of the mechanisms driving the response, and associated risks, accrue with advancing age. Clearly younger individuals who otherwise meet criteria for hypertension, like most of the cohort reported by Palatini et al., should be treated regardless of their orthostatic pressures. The unresolved question is how to manage those with isolated orthostatic hypertension.

We welcome the contribution by Palatini et al., which highlights the importance of recognizing orthostatic hypertension, and emphasizes the need for further research in this area. For example, mechanisms contributing to sympathetically mediated increases in blood pressure with standing deserve to be studied in more detail. Orthostatic hypertension has also been described in younger individuals suffering from other “hyperadrenergic” conditions such as postural tachycardia syndrome, which are arguably unrelated to hypertension. The clinical implication for autonomic specialists is that orthostatic testing is useful in detecting orthostatic hypotension and orthostatic hypertension, which both indicate altered autonomic cardiovascular control. However, even smaller changes in blood pressure may have prognostic implications.

## Data Availability

Not applicable.
